# A Synergistic Approach Combining Stable Carbon Isotope Ratio Analysis and Melissopalynology for the Authentication of Honey from Thailand

**DOI:** 10.3390/foods14223850

**Published:** 2025-11-11

**Authors:** Kunchit Judprasong, Chainarong Sinpoo, Sasiwimon Naksuriyawong, Kiattipong Kamdee, Sang-arun Meepho, Patcharin Phokasem, Chakrit Saengkorakot, Ratchai Fungklin, Nichtima Uapoonphol, Terd Disayathanoowat, Jeerawat Esor, Wisuwat Thongphichai, Kanokporn Boonsirichai

**Affiliations:** 1 Institute of Nutrition, Mahidol University, Nakhon Pathom 73170, Thailand; kunchit.jud@mahidol.ac.th (K.J.); meepho@gmail.com (S.-a.M.); wisuwat.tho@mahidol.ac.th (W.T.); 2 Office of Research Administration, Chiang Mai University, Chiang Mai 50200, Thailand; chainarong.s@cmu.ac.th (C.S.); patcharin.ph@cmu.ac.th (P.P.); 3 Department of Biology, Faculty of Science, Chiang Mai University, Chiang Mai 50200, Thailand; terd.dis@cmu.ac.th; 4 Nuclear Technology Service Center, Thailand Institute of Nuclear Technology (Public Organization), Nakhon Nayok 26120, Thailand; sasiwimon@tint.or.th (S.N.); ratchai@tint.or.th (R.F.); jeelawat@tint.or.th (J.E.); 5 Nuclear Technology Research and Development Center, Thailand Institute of Nuclear Technology (Public Organization), Nakhon Nayok 26120, Thailand; kiattipong@tint.or.th (K.K.); chakrit@tint.or.th (C.S.); nichtima@tint.or.th (N.U.); 6 Research Center of Deep Technology in Beekeeping and Bee Products for Sustainable Development Goals (SMART BEE SDGs), Chiang Mai University, Chiang Mai 50200, Thailand

**Keywords:** honey, stable carbon isotope, melissopalynology

## Abstract

Honey adulteration has long been a nuisance in local and international trade. Sugar syrup addition and false labeling of botanical origin have created a challenge in identifying fraudulent honey supplies and products. Stable carbon isotope ratio analysis (SCIRA) has been widely employed in honey authentication. While it is effective in identifying the addition of C4 plant-derived sugars, it does not provide information related to honey’s botanical source. This research investigated the combination of SCIRA and melissopalynology to provide a more robust assessment of honey integrity and showed that PCA analysis of δ^13^C together with sugar profiles could further improve the decision involving addition of sugar syrups. A total of 34 beekeeper honey samples were analyzed from 7 provinces of Thailand with a focus on longan honey. Twenty-four samples passed the δ^13^C criteria, exhibiting δ^13^C of bulk honey ranging from −28.53 ± 0.19‰ to −22.89 ± 0.08‰ and δ^13^C of extracted protein ranging from −29.30 ± 0.07‰ to −22.76 ± 0.03‰. Pollen profiling further eliminated honey of questionable and multifloral origins, yielding only eight samples that passed both criteria of being monofloral and not being adulterated with C4-derived sugars. These included six samples of longan honey and two honey samples of other botanical origins, yielding an overall passing rate of 23.5%. Our study showed that by combining SCIRA and melissopalynology, a robust determination of honey integrity could be achieved.

## 1. Introduction

Honey is a natural sweetener with bioactive properties, and it has been important for local and international trade across the globe [1]. A vast majority is produced from flower nectar by honeybees. It provides an important source of carbohydrates as it contains at least 60% fructose and glucose, and usually less than 5% sucrose, with small amounts of pollen, proteins, enzymes, vitamins, polyphenols, and organic acid [2,3]. As the market grows, consumers become more aware of the value of authentic honey with verifiable botanical origins, especially monofloral honey with unique flavor. In Thailand, honey is produced from many floral sources including longan, lychee, wildflower, and many others. Longan honey is a commercially important honey for both local consumption and international trade [4,5]. In 2019, Thailand honey export value rose to USD 19 million with an export amount of 7900 tons [6]. Therefore, determining product authenticity and verifying its botanical origin are of interest and have the potential to promote value addition of honey products.

Longan honey is derived from the nectar of the *Dimocarpus longan* L. It is a famous monofloral honey among consumers in East and Southeast Asia [5]. Thailand is one of the lead producers and exporters of longan honey, with the major production area in the Chiang Mai-Lamphun valley in the northern region [7,8]. Important international markets include China, Taiwan, and Japan. In addition to its characteristic smell, flavor, and natural sweetness, studies have shown that it exhibits interesting antioxidant and antimicrobial properties [5,9,10]. As a result, longan honey is often considered a premium product and is sold at a higher price than many other kinds of honey on the market. Due to its ability to achieve a higher price, the market is at risk of it being adulterated to obtain a more profitable sales margin. Although the extent of adulterated longan honey being sold or exported is not known, a previous study has demonstrated the existence of false claims regarding the authenticity of longan honey [11].

An authentication issue of concern is the addition of external sugar syrup to increase the volume of honey. The syrups often used by honey producers are sugarcane-derived, causing the sucrose content to exceed the standard level [12,13]. Sugarcane is considered a C4 plant with the Hatch–Slack photosynthesis pathway [14]. C4 plants such as sugarcane and maize can be differentiated from C3 plants, which form the majority of plant species within the Plant Kingdom, by their anatomy, physiology, and stable carbon isotope ratio [14,15,16,17]. Differences in stable carbon isotope ratio, especially the δ^13^C value, between C3 and C4 plants stem from their physiological differences in biological isotope fractionation related to the photosynthetic pathway. C3 plants typically exhibit δ^13^C values ranging from −30‰ to −22‰, while C4 plants typically have δ^13^C values of approximately −14‰ to −10‰ [18]. Consequently, δ^13^C analysis has been widely accepted as a method to detect exogenous C4 carbon in honey [19,20,21] and can be applied to longan honey and most other types of Thai honey from C3 botanical origin.

Botanical origin of honey is an important factor that influences buying decisions. Sources of nectar can impact on the appearance, texture, flavor, and physicochemical properties of honey [22]. To determine botanical origin, melissopalynological analysis is the method of choice [23,24]. Honey is considered monofloral when it contains a high percentage of pollen from a single source, typically greater than 45% [24]. It has been established that the pollen species with greater than 45% prevalence is the predominant pollen, those with 16–45% prevalence are the secondary pollen, those with 3–15.9% prevalence are important minority pollen, and those with less than 3% prevalence are the minority pollen [24].

This study investigated the efficacy of combining stable carbon isotope ratio analysis and melissopalynology to authenticate honey purity from undeclared carbon sources and to verify the botanical origin of honey. It covered a range of honey types with an emphasis on longan honey from northern provinces of Thailand and including a few honey samples of other botanical origins from other parts of the country. Combustible-module cavity ringdown spectroscopy (CM-CRDS) was used to establish the method to detect C4 contamination. CM-CRDS requires a lower cost of analysis, is simpler to operate, and has been proven in various studies to have a comparable precision and stability to the standard method, elemental analyzer–isotope ratio mass spectrometry (EA-IRMS) [25,26], making it practical for routine honey testing, especially in smaller laboratories or for on-site testing. In addition, pollen identification and counting were used to verify the nectar sources. We proposed that the two methods can be combined to detect fraudulent honey samples and can potentially be used in the screening of suppliers for the local honey industry in Thailand.

## 2. Materials and Methods

### 2.1. Honey Samples

The authentic honey was the raw longan honey stock obtained from a honey factory in Chiang Mai province, Thailand. The sample was analyzed by Intertek Food Services GmbH, Bremen, Germany, using EA/LC-IRMS to confirm authenticity (analysis report no. 2212210813). A total of 34 honey samples from various botanical sources were obtained from beekeepers with colonies of *Apis mellifera* L. on a voluntary submission basis. These included 1 sample of coffee blossom honey, 2 samples of sunflower honey, 2 samples of lychee honey, 3 samples of wildflower honey, and 26 samples of longan honey. These samples mostly came from bee farms in Chiang Mai. The rest of them came from Chiang Rai, Lamphun, Phrae, Nan, Saraburi, and Chumphon province of Thailand.

### 2.2. Adulterants

Adulterants used in this study came from commercial supplies. They included maltose syrup, brown rice syrup, molasses, golden syrup, invert sugar, high-fructose syrup 55%, cane syrup, corn syrup, and glucose syrup. For the deliberate adulteration experiment, syrups were added to the authentic honey samples at 2%, 5%, 10%, 20%, and 50% (*w*/*w*).

### 2.3. Sugar Profile Determination

The sugar profiles of the authentic honey sample and deliberately adulterated honey samples were analyzed for total sugar, glucose, fructose, sucrose, and maltose using high-performance liquid chromatography according to the AOAC 980.13 method [27].

### 2.4. Stable Carbon Isotope Analysis

Bulk honey and its protein fraction were prepared according to AOAC Method 998.12 C4 Plant Sugars in Honey [21]. Stable isotope ratio ^13^C/^12^C was measured using combustion module–cavity ring down spectroscopy (CM-CRDS) (Picarro G2131-I, Picarro Inc., Santa Clara, CA, USA). The carbon isotope ratios were normalized against USGS61 and USGS62 reference materials. For measurements using isotope ratio mass spectrometry (IRMS, Delta V Advantage, Thermo Fisher Scientific, Waltham, MA, USA), the isotope ratios were normalized against USGS40 and USGS41a reference materials. δ^13^C was calculated according to the following equation in parts per thousand (‰) with reference to Vienna Pee Dee Belemine (VPDB) standard. Triplicate measurements were performed for each sample.

δ^13^C = [(^13^C/^12^C)_sample_/(^13^C/^12^C)_VPDB_ − 1] × 1000, (1)

### 2.5. Melissopalynology

Pollen preparation and analysis were performed according to Louveaux et al., 1978 [24]. A total of 5 g of honey was mixed with 5 mL of sterilized distilled water and centrifuged at 6000 rpm for 30 min. The precipitate was diluted with 100 µL of distilled water and counted using hemocytometers under a microscope with 400× magnification. For each honey sample, quantitative pollen analysis was performed using three independent replicate slides. A minimum of 500 pollen grains was counted per slide, or the entire area scanned if fewer grains were present [24]. Pollens were identified based on comparison with reference slides made from identified plants. Their prevalence was classified according to Louveaux et al., 1978 [24].

### 2.6. Statistical Analysis

One-way ANOVA was performed to compare the means among groups of samples. Differences were considered statistically significant if *p* < 0.05. Homogeneity of variances was tested to satisfy ANOVA assumptions. When ANOVA indicated significant differences, Tukey’s Honestly Significant Difference (HSD) post hoc test was conducted to compare all pairs of group means while controlling the Type I error rate. Principal component analysis (PCA) was performed to classify adulterated honey. IBM SPSS (Chicago, IL, USA) Version 19.0.0 was used for both analyses.

## 3. Results and Discussion

### 3.1. δ^13^C of Authentic Honey

δ^13^C measurements of an authentic sample of longan honey obtained from Chiang Mai province, Thailand, were carried out using EA/LC-IRMS, EA-IRMS, and CM-CRDS methods (Table 1). The average δ^13^C of bulk honey (δ^13^C_H_) was −26.40 ± 0.13‰, and that of honey protein (δ^13^C_P_) was −25.75 ± 0.22‰. The three methods did not yield significant differences in δ^13^C_H_ (*p* > 0.05), but significant differences in δ^13^C_P_ were found among the three samples. However, δ^13^C values measured using CM-CRDS and EA/LC-IRMS were not statistically different. The significant differences in δ^13^C_P_ among the three methods could stem from the fact that the protein content in honey is very low (~0.2–0.6%) [28]. Thus, protein extraction could be prone to contamination with residual sugars, organic acids, and other compounds that might co-precipitate during extraction. As a result, sufficient amounts of starting materials are needed, and proper handling of samples is crucial. In addition, proper drying of protein extract must be ensured because retained moisture can interfere with CM-CRDS measurements and affect isotopic accuracy, as water vapor absorbs in similar spectral regions as CO_2_ [29].

In terms of authenticity, the sample being investigated was categorized as authentic honey, as its δ^13^C_H_ was within the range of δ^13^C for C3 plants (<−22.00‰) [16,17], and the difference between δ^13^C_P_ and δ^13^C_H_ (∆δ^13^C_P-H_) was less than 1.00‰ [30,31] (Table 1). These observations were in accordance with a previous report of Thai honey with δ^13^C_H_ in the range of −29.1‰ to −23.1‰ [30], in compliance with the AOAC guideline of −32.0‰ to −23.5‰ [20,21], and in similar ranges to previously reported data from other countries [30,31,32,33,34].

### 3.2. Comparison Between CM-CRDS and EA-IRMS for δ^13^C Measurements

Although EA-IRMS is an official AOAC method for determining the presence of C4 sugar in honey [21], advanced techniques are also available, including LC-IRMS and ^1^H-NMR. While EA-IRMS measures the bulk isotopic average of the sample, LC-IRMS separates the sugar composition and individually measures their isotopic characteristics. EA-IRMS application is limited to the detection of added C4 sugars with a sensitivity of approximately 7% [21]. It cannot detect added C3 sugars, which share the same δ^13^C range with most honey products. LC-IRMS shows high sensitivity and can detect both C3 and C4 sugar addition at the levels as low as 3–5% [33], but it requires complex instrumentation and a highly skilled operator. On the other hand, ^1^H-NMR provides a chemical fingerprint of the sample, can detect C3 and C4 sugar adulteration, and can be used to determine geographical and botanical origins [35]. Interpretation of ^1^H-NMR data requires a reference database, which can influence the interpretation itself. It offers a fast and simple method of adulteration detection. The limitation lies in its high investment cost and lower sensitivity for sugar adulteration.

**Figure 1:**
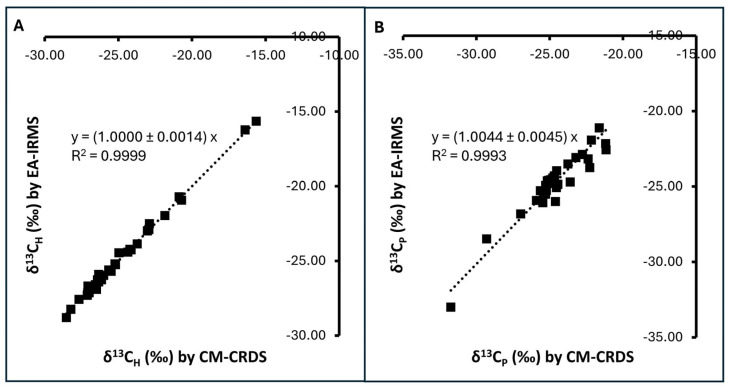
CM-CRDS and EA-IRMS measurements of 34 honey samples exhibiting a linear relationship: (**A**) δ^13^C of bulk honey (δ^13^C_H_); (**B**) δ^13^C of honey protein (δ^13^C_P_). The equations show the slope ± standard error for y = *a*x. Regression analysis yielded *p* < 0.00001 for both (**A**) and (**B**), indicating significant relationships.

CM-CRDS is an alternative technique. Its principle of adulteration detection relies on δ^13^C determination of bulk samples, similar to EA-IRMS with similar limitations [32,36]. However, it requires less capital investment and does not need a highly skilled operator. Previous studies have shown that CM-CRDS is robust and can be used as an alternative to EA-IRMS [30,32,36]. To confirm its robustness against EA-IRMS, 34 Thai honey samples were analyzed for their δ^13^C_H_ and δ^13^C_P_ using both CM-CRDS and EA-IRMS techniques. CM-CRDS and EA-IRMS yielded comparable results of δ^13^C values for both bulk honey samples and the protein extracts (Figure 1). δ^13^C_H_ was found in the range of −28.80‰ to −15.65‰ using EA-IRMS and −28.53‰ to −15.64‰ using CM-CRDS. δ^13^C_P_ was found in the range of −33.01‰ to −21.12‰ using EA-IRMS and −33.75‰ to −21.15‰ using CM-CRDS. Regression analysis showed that the δ^13^C_H_ and δ^13^C_P_ values measured using the two methods formed a linear relationship with the slope values close to 1 (R^2^ > 0.999) (Figure 1). The regression *p* values were less than 0.00001 for both techniques, indicating significant relationships (regression statistics can be found in Tables S1 and S2). Reproducibility testing of CM-CRDS was performed on a honey sample with nine separate preparations and measurements, yielding RSDs of less than 1% (Table S3). These observations were in agreement with other studies where EA-IRMS and CM-CRDS were found to be comparable [30,32,37], supporting the robustness of CM-CRDS and indicating that it can be used as an alternative to EA-IRMS. It is to be noted that regression analyses of δ^13^C_P_ data yielded a larger residual sum of squares than δ^13^C_H_ data (Tables S1 and S2), indicating greater departure of data points from the predicted values, as seen in Figure 1B. This observation underlines our previous discussion regarding the reported limitation of honey protein extract preparation and interference of sample moisture in CM-CRDS measurements. Although some limitations exist, CM-CRDS results appeared robust and comparable to EA-IRMS. Therefore, it was chosen as the method of choice in subsequent analyses.

### 3.3. Deliberate Adulteration of Authentic Honey

#### 3.3.1. δ^13^C of Adulterants

To determine the ability of CM-CRDS to detect added C4 sugar in honey samples, sugar syrups from C3 and C4 sources were added to authentic honey at varying concentrations. δ^13^C, which indicates the relative abundance of ^13^C in comparison to ^12^C, was used to identify the carbon sources of the syrups and honey samples. The photosynthetic pathway of C3 plants uses the Calvin Cycle to directly fix CO_2_ in the mesophyll cells to form 3-phosphoglyceric acid, a 3-carbon compound, while in C4 plants, CO_2_ is initially fixed in mesophyll cells to form oxaloacetate, a 4-carbon compound [38]. Then, oxaloacetate is converted to malate and transported into the bundle sheath cells where Calvin Cycle fixation occurs [39]. These physiological differences resulted in differing distributions of natural carbon isotopes between C3 and C4 plants. C3 plants exhibit δ^13^C in the range of −32‰ to −22‰, whereas C4 plants exhibit δ^13^C in the range of −16‰ to −8‰ [18].

In this study, C3 and C4-derived syrups were used in the deliberate adulteration experiment. Commercial syrups derived from rice, barley, and cassava represented adulterants from C3 sugar sources, while those derived from sugarcane and corn represented adulterants from C4 sugar sources. Their δ^13^C values, as shown in Table 2, corresponded well to their source of origins except for maltose syrup. δ^13^C values of cane syrup, golden syrup, and molasses ranged from −13.23 ± 0.10‰ to −12.78 ± 0.29‰, which corresponded well to the previously reported range of −14‰ to −10‰ for sugarcane-derived syrup [34]. The δ^13^C value of corn syrup was −11.76 ± 0.09‰, which was slightly lower than the previously reported range of −11.5‰ to −9.7‰ [34,40] but still within the C4 δ^13^C values [18]. The δ^13^C value of rice syrup was −28.29 ± 0.20‰, which was slightly lower than the previously reported range of −28‰ to −22‰ [41,42] but still corresponded to the C3 δ^13^C values [18]. High-fructose syrup and glucose syrup derived from cassava showed δ^13^C values of −26.92 ± 0.32‰ and −27.20 ± 0.21‰, respectively, which were within the previously reported range of −33‰ to −24‰ [15]. Invert sugar derived from sugar beet exhibited δ^13^C values of −27.69 ± 0.29‰, which corresponded well to the previously reported range of −30‰ to −24‰ for sugar beet [41]. The slight discrepancies that were found for corn syrup and rice syrups could be due to differences in geographical origins and agricultural practices, which are known to influence δ^13^C values [43,44,45,46,47]. On the other hand, maltose syrup, which was labeled as deriving from rice and barley, showed a δ^13^C value of −18.83 ± 0.21‰ which lied in between the C3 and C4 δ^13^C ranges, suggesting a mixture of C3 and C4 carbon sources. Rice syrup δ^13^C values were previously reported in the range of −28‰ to −22‰ [48], and barley malt extract δ^13^C values were reported to be below −24.3‰ [49], well within the range of δ^13^C for C3 plants. Therefore, the deviation of the δ^13^C of maltose syrup in this study would indicate the presence of undeclared C4 carbon in this syrup sample, which can be found in some commercial products [49].

#### 3.3.2. Sugar Profile of Authentic Honey

Like most honey, the sugar profile of the authentic longan honey used in this study was primarily dominated by reducing sugar, namely fructose and glucose. Its total sugar content was 73.6% by weight, comprising 37.4% fructose, 29.7% glucose, 4.0% maltose, and 2.5% sucrose. These observations were close to the reported range of honey sugar profiles from other botanical sources. Waworuntu (2024) [50] reported the total sugar content and reducing sugar content of honey from four botanical sources to be in the range of 78.40–80.30% and 64.4–65.02%, respectively. Fructose content may range from 35 to over 40%, glucose content from a little below 30% to close to 40%, while maltose and sucrose contents are typically below 5% [51,52,53].

#### 3.3.3. Sugar Levels of Deliberately Adulterated Honey

**Figure 2:**
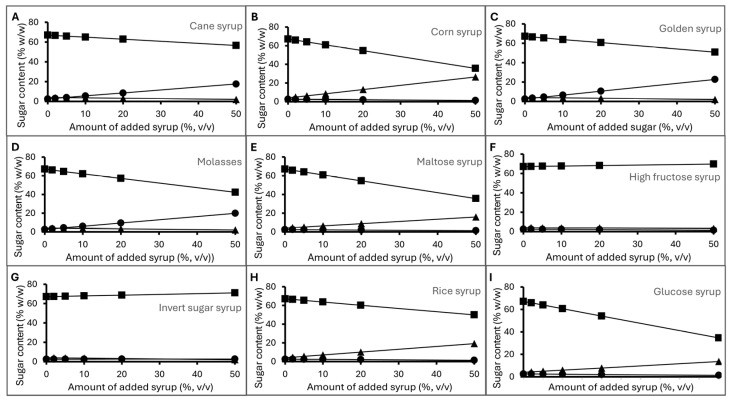
Sugar content of honey samples intentionally adulterated with (**A**) cane syrup, (**B**) corn syrup, (**C**) golden syrup, (**D**) molasses, (**E**) maltose syrup, (**F**) high-fructose syrup, (**G**) invert sugar syrup, (**H**) rice syrup, (**I**) glucose syrup. Markers represent reducing sugar (fructose and glucose) content (square), sucrose content (circle), and maltose content (triangle).

The additions of syrups as external sugar sources at 5%, 10%, 20%, and 50% may or may not significantly change the sugar profile of longan honey detected using HPLC (Figure 2). Cane syrup, golden syrup, and molasses from sugarcane contained high levels of sucrose. When added at 10%, it caused the sucrose level in the mixture to rise above 5% (Figure 2A,C,D), which is the maximum level allowed by the CODEX Standard for honey [54], while the reducing sugar levels were still above 60%. High-fructose syrup and invert sugar even added at 50% by weight still kept the sucrose level below 5% and the reducing sugar above 60% (Figure 2F,G). On the other hand, the addition of corn syrup, maltose syrup, rice syrup, and glucose syrup at increasing levels caused the reducing sugar contents in the honey mix to decrease and the maltose level to increase (Figure 2B,E,H,I). At 10%, their addition led to a rise of maltose levels to above 5%. As a result, sugar content can be used to detect external sugar, with some limitations. It can detect the addition of cane-derived sugar when the sucrose level rises above 5% and glucose/maltose-based sugar sources when maltose becomes higher than 5%. However, it cannot detect adulteration at a lower level or adulteration with fructose-based syrup or invert sugar.

#### 3.3.4. δ^13^C of Deliberately Adulterated Honey

**Figure 3:**
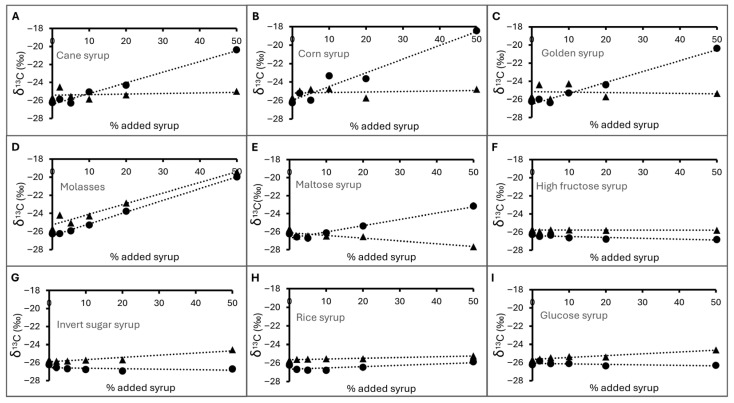
ẟ^13^C of longan honey samples deliberately adulterated with (**A**) cane syrup, (**B**) corn syrup, (**C**) golden syrup, (**D**) molasses, (**E**) maltose syrup, (**F**) high-fructose syrup, (**G**) invert sugar syrup, (**H**) rice syrup, (**I**) glucose syrup. Markers represent δ^13^C_H_ (circles) and δ^13^C_P_ (triangle). Dotted lines indicated the measurement trends only.

Figure 3 shows the changes in the δ^13^C of longan honey deliberately adulterated with various sources of external sugar. The longan honey without any added sugar syrup showed a δ^13^C value of −26.26 ± 0.09‰ measured using CM-CRDS technique in this study. This value was close to −26.43‰, the δ^13^C value measured by EA/LC-IRMS (Table 1), and in the same range as previously reported values for pure longan honey from Taiwan and Thailand (−26.57‰to −24.53‰) [55]. Rises in δ^13^C_H_ were observed when they were adulterated with syrup that came from a C4 source including sugarcane and corn (cane syrup, corn syrup, golden syrup, molasses: Figure 3A–D), as these sugar sources exhibited significantly higher δ^13^C values than authentic longan honey (see discussion above). Interestingly, samples adulterated with maltose syrup also showed a rise in δ^13^C_H_ (Figure 3E). As mentioned earlier, this sample of maltose syrup showed a δ^13^C value that indicated the presence of both C3 and C4 sugar sources. As a result, its addition into longan honey would cause the observed increases in the δ^13^C_H_ of the mixture. On the other hand, the addition of syrup from a C3 carbon source, including cassava-derived high-fructose and glucose syrups, sugar beet invert sugar, and rice syrup, did not result in a significant rise in δ^13^C_H_ (Figure 3F–I).

To determine honey adulteration, δ^13^C values of honey protein (δ^13^C_P_) are also determined to use as an internal comparison for the δ^13^C value of bulk honey samples (δ^13^C_H_). Honey contains proteins in small quantities comprising several enzymes and pollen proteins [56], which will influence its δ^13^C_P_ and δ^13^C_H_ values. As the majority of flowering plants utilize the C3 photosynthetic pathway, the δ^13^C of honey and its proteins are often within the range of C3 plants. The addition of external C4 sugars will affect δ^13^C_H_ and theoretically will not affect δ^13^C_P_. Therefore, δ^13^C_P_ is used as an internal reference. Departures of δ^13^C_H_ from δ^13^C_P_ will indicate the presence of an external C4 carbon source. The use of δ^13^C_P_ as an internal reference has been widely accepted and utilized commercially [21].

Extracted protein from longan honey had δ^13^C_P_ values of −25.68 ± 0.08‰, measured using the CM-CRDS technique, and −25.57‰ measured using EA/LC IRMS (Table 1). δ^13^C_P_ from deliberately adulterated honey samples mostly remained statistically unchanged, showing an average value of −25.4 ± 0.8‰. This observation pertained to honey samples adulterated with cane syrup, corn syrup, golden syrup, maltose syrup, high-fructose syrup, invert sugar syrup, and glucose syrup. This is due to the fact that sugar syrups are normally made of sugar and water as the main ingredients without a protein source. As a result, all proteins obtained from the extraction process had a honey origin. On the other hand, extracted proteins from longan honey adulterated with molasses showed a rise in δ^13^C_P_. Although molasses was not supposed to contain any proteins, a rise and high correlations between δ^13^C_H_ and δ^13^C_P_ of the adulterated samples were observed. It was possible that part of molasses sugars might have coprecipitated with the proteins, causing the contamination of molasses carbon in the protein fraction. Interestingly, the δ^13^C_P_ of molasses-adulterated honey eventually rose above the C3 δ^13^C upper range of −22.00‰. This observation strongly supports the molasses–honey protein coprecipitation hypothesis and might indicate a limitation of the technique.

Differences between δ^13^C_H_ and δ^13^C_P_ can be used to determine adulteration by C4 sugar sources. White and Winters (1989) [28] proposed to use differences of 1‰ or more as the criteria for honey adulterated with C4 sugar. Considering this, our experiments could potentially detect the addition of corn syrup at 5%; golden syrup, invert sugar syrup, and molasses at 10%; cane syrup and maltose syrup at 20%; and high-fructose syrup and glucose at 50% and above. The technique appeared not to detect the addition of rice syrup reliably.

#### 3.3.5. Chemometric Analysis of Deliberately Adulterated Honey

**Figure 4:**
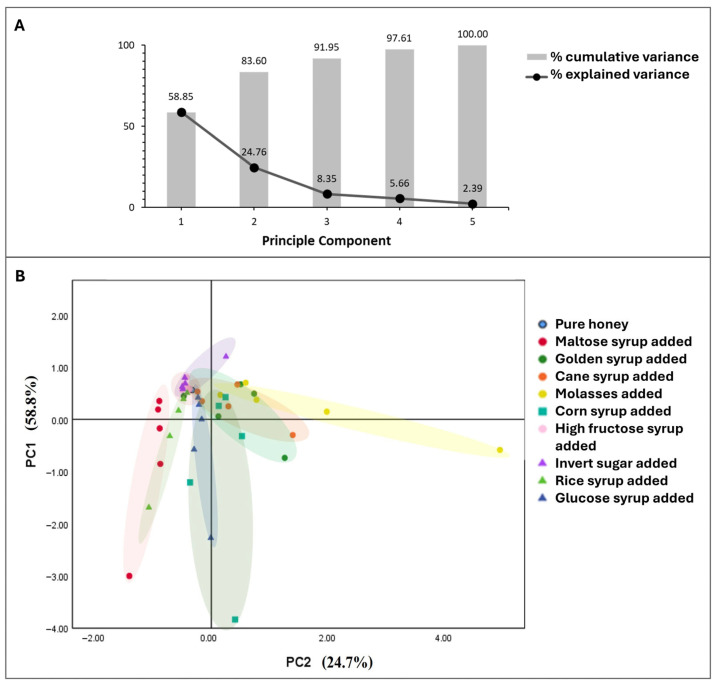
Principal component analysis showing (**A**) explained variance for each principal component and cumulative variance with each additional component, (**B**) PCA score plot of honey samples deliberately adulterated with maltose syrup, golden syrup, cane syrup, molasses, corn syrup, high-fructose corn syrup, invert sugar, rice syrup, and glucose syrup.

Principal component analysis (PCA) has been used in a few studies to determine honey adulteration [52,53]. In this study, PCA was applied to five factors related to honey properties, including δ^13^C_H_, δ^13^C_P_, and amounts of fructose, glucose, and maltose in honey samples. Only these three sugars were considered together with δ^13^C_H_, δ^13^C_P_ because the Kaiser–Meyer–Olkin Measure of Sampling Adequacy indicated better suitability for factor analysis (0.69) compared to including sucrose (0.53). Bartlett’s Test of Sphericity was significant (chi-square = 126.18, *p* < 0.001), indicating a strong relationship among the five variables. The first two eigenvalues (PC1 and PC2) equaled 2.94 and 1.24, which represented 83.6% of the total variability (Figure 4, Table S4). Component 1 (PC1) comprised the fructose level, the glucose level, and the maltose level, while Component 2 (PC2) comprised δ^13^C_H_ and δ^13^C_P_. The PCA score plot (Figure 4) placed the authentic longan honey in quadrant II (positive PC1 and negative PC2).

Greater than 10% adulteration caused most samples to be placed outside the authentic honey quadrant. According to Figure 4, the PCA plot shows that honey samples with ≥10% sugar adulteration by C4-plant-derived adulterants like cane syrup, molasses, corn syrup, and golden syrup could be easily distinguished. However, a limitation of PCA application remained, as it was unable to display the adulteration of honey with C3-plant-derived adulterants like high-fructose corn syrup and invert sugar at concentrations lower than 20%. Samples adulterated at 20% or lower with high-fructose corn syrup and invert sugar still fell within the “authentic honey” region.

The PCA plot in Figure 4 reveals distinct distribution trends for different adulterants in the honey samples, with clear separation of C4 sugar-adulterated samples and partial overlap for C3 sugar-adulterated samples. Adding different adulterants showed unique isotopic and sugar profile signatures, which aided in the identification of adulteration patterns. Considering different clusters along PC1 which comprised sugar levels of glucose, fructose, and maltose, adulterating honey with more corn syrup, maltose syrup, glucose syrup, or rice syrup led to a large change in individual sugar content presented in the samples (cluster widely spread along PC1 in the lower left quadrant with higher negative PC1 values compared to other adulterants).

Different distribution trends were found for PC2. Including molasses in honey samples led to a significant shift of δ^13^C toward less negative values compared to pure honey (cluster appeared widely along PC2 in the upper-right quadrant with much higher positive PC2 values than other adulterants). Adding more than 10% of cane syrup or golden syrup also caused an obvious reduction in the δ^13^C values of the honey samples; however, to a smaller extent than molasses (a moderate spread along PC2). Glucose syrup, rice syrup, high-fructose syrup, invert sugar, and maltose showed a low influence on the shift in δ^13^C values (narrow cluster spread along PC2). Clusters of samples with <10% rice syrup or glucose syrup fell within the same quadrant (upper-left) as pure honey, and the values remained closer to the authentic region, making the identification ambiguous.

### 3.4. Authentication of Thai Honey

#### 3.4.1. ẟ^13^C of Thai Honey

**Figure 5:**
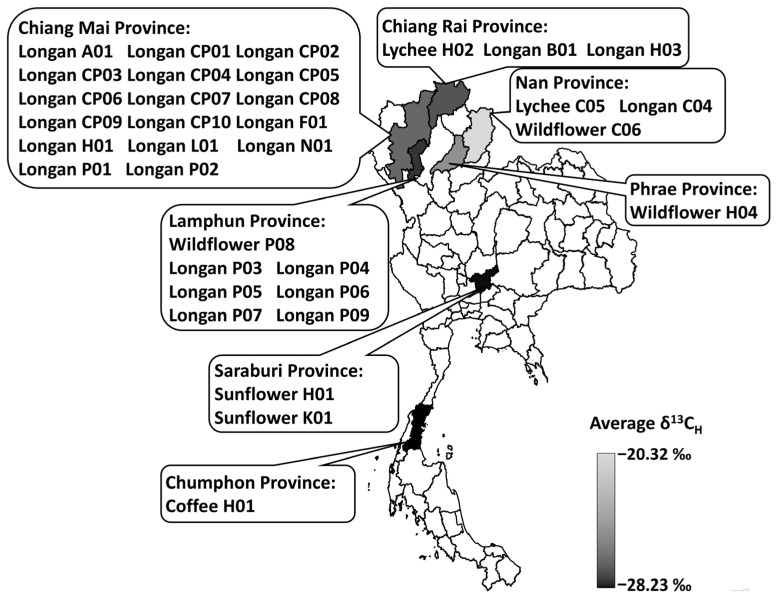
Map of the provincial origins of honey samples and the average δ^13^C_H_. Thirty-four samples were collected from Chiang Mai, Chiang Rai, Chumphon, Lamphun, Nan, Phrae and Saraburi, Thailand. Color intensities represent the levels of provincial-averaged δ^13^C_H_.

Thirty-four honey samples were collected from Chiang Mai, Chiang Rai, Chumphon, Lamphun, Nan, Phrae, and Saraburi provinces, Thailand, on a voluntary submission basis by beekeepers. However, the campaign was targeted to longan honey, as it was the focus of this study. The obtained samples consisted of 26 samples of longan honey, 2 samples of lychee honey, 3 samples of wildflower honey, 2 samples of sunflower honey, and 1 sample of coffee blossom honey. Most samples were from northern provinces, except for the sunflower honey samples, which were from the central region, and the coffee blossom honey, which was from Southern Thailand (Figure 5). Northern Thailand is an important production region of longan fruits (*Dimocarpus longan* L.) for internal consumption, exporting over 80% of Thai longan fruits [57], which are highly dependent on bees for pollination. Apiculture and longan honey production are gaining in economic importance, and premium longan honey is in high demand for export. In this study, only a few samples of wildflower, lychee, sunflower, and coffee blossom honey were included. Therefore, they were not considered good representatives of their kind. The purpose of their inclusion in this study was to provide a more generalized view of the Thai honey landscape in addition to the repertoire of longan honey.

Bulk honey and honey proteins were examined for their ẟ^13^C values. A range of −28.53‰ to −15.64‰ was observed for δ^13^C_H_ and −31.75‰ to −21.15 to‰ for δ^13^C_P_ (Table 3 and Figure 5). The δ^13^C_H_ values of longan honey were found in similar ranges as other honey samples: −27.68‰ to −16.40‰ for longan honey and −28.53‰ to −15.64‰ for other honeys. When Grubb’s test was applied to all 34 honey samples (Table 3), the δ^13^C_H_ of Wildflower C06 at −15.64‰ was identified as an outlier (*p* < 0.05). When applied to the 26 longan honey samples, the δ^13^C_H_ of Longan CP04 at −16.40‰ was identified as an outlier (*p* < 0.05). By eliminating the outliers, the δ^13^C_H_ range of the longan honey became −27.68‰ to −20.80‰. When comparing this range to the δ^13^C_H_ range of C3 plants, it was apparent that a few of longan honey and other honey samples contained C4 carbon sources.

When comparing the δ^13^C_H_ ranges of longan and other honey samples to that of C3 plants (−32‰ to −22‰ [15,17,18]), it was apparent that a few longan honey samples, as well as a few other honey samples, contained C4 carbon sources. To determine the authenticity of the honey samples, first, their δ^13^C_H_ values were considered in comparison with the upper limit of the δ^13^C value of C3 plants. Acceptable samples must exhibit a δ^13^C value not exceeding −22.00‰ (Table 3). This caused three longan honey samples (CP04 CP07 and N01) to be rejected, as well as wildflower honey C06 and lychee honey C05. Next, δ^13^C differences between the protein fraction and bulk honey (δ^13^C_P_–δ^13^C_H_ or ∆δ^13^C_P-H_) were applied, with acceptable samples having ∆δ^13^C_P-H_ greater than −1.00‰ [28]. This criterion was successfully used in other previous reports including Kamdee et al. (2023) [30]. When applied to the honey samples in this study, two additional longan honey samples (CP01 and P02) as well as wildflower H04 and coffee H01 were rejected.

A discrepancy was observed for sample Longan N01; its δ^13^C_H_ was −21.84 ± 0.04‰ (above −22.00‰), but its ∆δ^13^C_P-H_ value was −0.53 (above −1.00‰). We rejected this sample, as its δ^13^C_H_ suggested the presence of C4 sugars. The passing ∆δ^13^C_P-H_ value was caused by a rather high δ^13^C_H_ of −22.37 ± 0.16‰. An explanation could be carried-over sugars during protein extract preparation or that the bee colony was also fed with cane sugars in addition to natural foraging, resulting in a small difference between δ^13^C_P_ and δ^13^C_H_. Another discrepancy was observed for Longan C04, where its δ^13^C_H_ was −24.36 ± 0.15‰ (below the criterion of −22.00‰), but its δ^13^C_P_ was −21.18 ± 0.13‰ (above the criterion of −22.00‰), and its ∆δ^13^C_P-H_ was 3.18 (above the criterion of −1.00‰). Using the criteria described above, this sample would appear to pass as acceptable. However, its δ^13^C_P_ made the decision ambiguous. This unexpectedly high δ^13^C_P_ could not be explained easily and might demonstrate limitations of protein extract preparation and/or CM-CRDS measurements, as discussed above. Therefore, it was decided to reject this sample.

**Figure 6:**
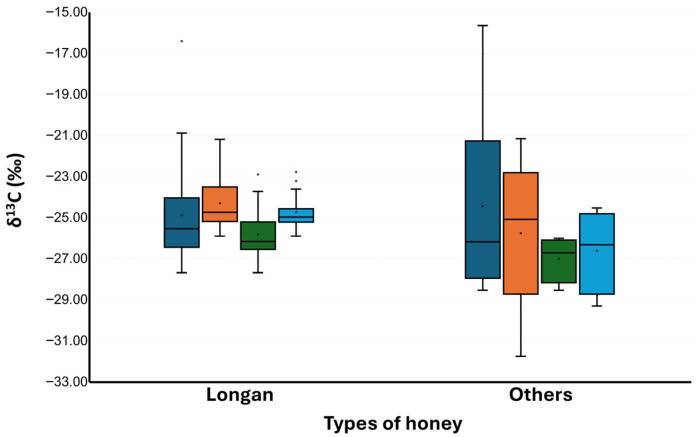
ẟ^13^C_H_ and ẟ^13^C_P_ of longan honey and other types of honey. Dark blue bars represent ẟ^13^C_H_ of collected samples. Orange bars represent ẟ^13^C_P_ of collected samples. Green bars represent ẟ^13^C_H_ of samples that passed the ẟ^13^C criteria. Light blue bars represent ẟ^13^C_P_ of samples that passed the ẟ^13^C criteria. Mean values are shown as black dots within the bar graphs; median values are shown as black horizontal lines within bar graphs.

At this point, 20 out of 26 longan honey samples (76.9%) passed the δ^13^C criteria. The overall passing rate for the 34 honey samples was 24 out of 34, or 70.6%. The δ^13^C_H_ range of acceptable honey samples was −28.53 ± 0.19‰ to −22.89 ± 0.08‰, with δ^13^C_P_ of −29.30 ± 0.07‰ to −22.76 ± 0.03‰. Acceptable longan honeys exhibited a δ^13^C_H_ range of −27.68 ± 0.05‰ to −22.89 ± 0.08‰ and δ^13^C_P_ of −25.90± 0.14‰ to −21.18 ± 0.13‰. Other kinds of honey exhibited a δ^13^C_H_ range of −28.53 ± 0.19‰ to −26.00 ± 0.05‰ and δ^13^C_P_ of −29.30± 0.07‰ to −24.52 ± 0.05‰ (Figure 6). The observed ranges, especially of δ^13^C_H_, were almost in the same range as that previously reported for Thai honey, which ranged from to −29.1‰ to −23.1‰ [30].

#### 3.4.2. Pollen Composition of Thai Honey

**Figure 7:**
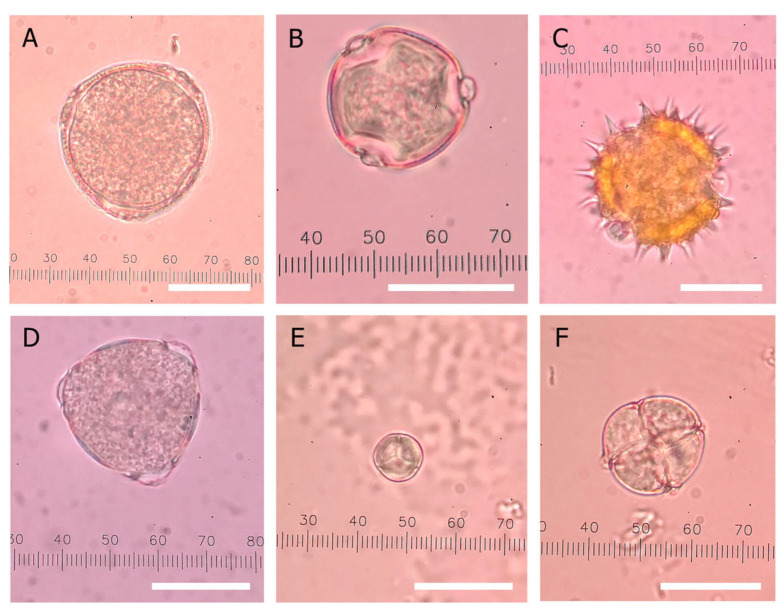
Examples of the major pollen found in this study. Pollen grains of (**A**) genus *Coffea*, the coffee genus, (**B**) *Dimocarpus longan* Lour., the longan tree, (**C**) *Helianthus annuus*, the sunflower plant, (**D**) *Litchi chinensis*, the lychee tree, (**E**) *Mimosa pudica* L., the sensitive plant, and (**F**) *Mimosa pigra* L., the catclaw mimosa plant. Each white scale bar corresponds to 20 μm in length.

The 34 honey samples were examined for their pollen composition to verify the botanical origin. All of them carried pollen from multiple plant species, with at least 12 pollen species identified in this study (Table 4). The six most prevalent pollens were of *Coffea* genus, *Dimocarpus longan* Lour., *Helianthus annuus*, *Litchi chinensis*, *Mimosa pudica* L., and *Mimosa pigra* L. (Figure 7). Pollens were classified based on their prevalence in honey as predominant (>45%), secondary (16–45%), minor (3–15.9%), and including (<3%) according to the method proposed by Louveaux et al. (1978) [24]. Thirty samples contained pollen that corresponded with the claimed plant origin as either the predominant or the secondary pollen (>16% prevalence), indicating their association with the claimed origin. When considering the longan honey group, 4 of 26 samples (longan A01, CP09, F01, N01) failed to carry *Dimocarpus longan* Lour. pollen at a significant level (>16%), indicating false claims of origin. For the other 22 longan samples, *Dimocarpus longan* Lour. pollen was present at 18–66% (Table 4). Other pollens found in the longan honey samples included those of *Mimosa pudica*, *Mimosa pigra*, *Leucaena leucocephala* L., Salicaceae, and *Bidens pilosa* L., with *Mimosa pudica* pollen found in all samples except for one (longan CP10). *Mimosa pudica* is a weed species that can be found across Thailand, including in longan orchards. Bees foraging in longan orchards would also be exposed to *Mimosa pudica* L. weeds, hence their presence at significant levels in the pollen content of most longan honey samples.

In Thailand, the longan flowering season typically occurs once per year, from February to April, depending on regional climatic conditions and orchard management [58]. During the late flowering or dry season, nectar availability can be substantially reduced, leading beekeepers to supplement colonies with sucrose syrup to maintain bee health and productivity [59,60]. This practice can result in honey with diluted floral markers and lower pollen representation from the primary source plant, even when the apiaries are located within longan orchards. In areas where longan orchards are interspersed with other crops (e.g., common flowering weeds like *Mimosa pudica* L.), bees will prioritize the most rewarding nectar source. When longan nectar is scarce, bees will forage widely, collecting both nectar and pollen from alternative sources. This mixed foraging pattern directly results in a lower proportion of *Dimocarpus longan* L. pollen in the final product. Moreover, variations in pollen content may also arise from differences in foraging behavior, local vegetation diversity, and post-harvest blending practices.

When the rest of honey samples are considered, samples of lychee honey, sunflower honey, and coffee blossom honey all had their corresponding pollen species present at greater than 16%, indicating their association with the claimed botanical origin (Table 4). *Litchi chinensis* pollen was present at 25–26% in lychee honey samples, while *Helianthus annuus* was present at 37–88% in sunflower honey samples. The single coffee blossom honey sample in this study contained pollens of *Coffea* genus at 93% prevalence. On the other hand, the wildflower honey samples in our study contained pollen from a few plant species. Interestingly, *Mimosa pudica* L. showed a high prevalence in these samples, being found at 61–81%. Other pollens found came from *Dimocarpus longan* L., *Bidens pilosa* L., and the Fabaceae family. This observation confirmed the above discussion that *Mimosa pudica* L. is one of the prevalent weed species in Thailand.

To be considered as monofloral honey, the criterion of the predominant pollen was applied, where pollen species of the claimed origin must be present at levels greater than 45%. Considering this, only 8 of the 26 longan samples could pass as monofloral longan honey; these were Longan C04, CP02, CP03, CP04, CP10, L01, P06, P09. These constituted 30.7% of the longan honey samples in this study. This low percentage might indicate that longan pollen was not abundantly present in the nectar, and a correction factor should be applied to account for the low pollen recovery rate. This correction factor is known as the pollen coefficient in melissopalynology. The pollen coefficient for *Dimocarpus longan* L. has not been reported previously. However, a study by Tangtragoon et al. [11] reported greater than 70% prevalence of *Dimocarpus longan* L. pollen found in 13 out of 19 monofloral longan honey samples. Thus, pollen abundance in the nectar might not be an issue for *Dimocarpus longan* L., and a pollen coefficient might not be needed. Nonetheless, a formal study to confirm this observation should be conducted.

When the monofloral honey criterion was applied to the rest of the honey samples in this study, it was found that Sunflower Honey K01 and Coffee Blossom Honey H01 could be identified as monofloral honey, having the corresponding pollen at greater than 45% prevalence (88% and 93%, respectively). The three wildflower honey samples contained *Mimosa pudica* L. as the predominant pollen and could be labeled as *Mimosa pudica* monofloral honey. However, *Mimosa pudica* L. is a weed species and has not gained recognition as a bee-foraging species, nor its honey as a monofloral honey. Therefore, “wildflower honey” labelling would likely be more appropriate commercially.

#### 3.4.3. Authentication Consideration

Many studies of honey authentication considered only a single aspect of authentication, being C4 sugar adulteration, geographical origin, or botanical origin. This study took both C4 sugar adulteration and botanical claim into consideration. δ^13^C analysis was first applied to eliminate samples with possible C4 sugar addition. Then, melissopalynology was applied to keep only monofloral honey samples.

Of the 34 honey samples, 24 samples were accepted based on their δ^13^C values as samples without C4 sugar adulteration (Table 5). Of these, eight samples could be considered monofloral honey with correct botanical claim. A correlation between the two sets of criteria was not observed, as they are independent technically. The samples that “passed” were six longan honey samples (CP02, CP03, CP10, L01, P06 and P09), one sunflower honey sample (K01), and one wildflower honey sample (P08) (Table 5), although *Mimosa pudica* honey was labeled as wildflower honey, as discussed above. Considering each set of criteria separately, the passing rate for δ^13^C analysis was 24 out of 34, or 70.6%, and for melissopalynology, it was 13 out of 34, or 38.2%. A low passing rate under melissopalynology criteria might come from the beekeepers’ practice of naming their honey according to the major crops in orchards where they place their bee colonies. Since pollen analysis is not routinely practiced, honey products were not subjected to proper quality control regarding pollen composition. However, for premium monofloral honey products, especially monofloral longan honey, it is recommended that melissopalynology is performed to ensure quality and proper claim of the botanical origin.

Implementing δ^13^C and melissopalynology criteria together resulted in a more stringent consideration than either set of criteria alone. Together, they yielded a passing rate of 8 out of 34, or 23.5% (Table 5). When the longan honey samples were compared with the rest of the honey samples, a passing rate of 23.1% was observed for longan honey, and a 25.0% passing rate was observed for the other kinds of honey. It appeared that the final passing rates did not differ greatly between longan honey and the other honey, as well as among the longan honey group, the other honey group, and the entire sample set of honey together. It should be noted that other kinds of honey besides longan honey were underrepresented in this study. The comparison of their passing rates must be interpreted with caution.

## 4. Conclusions

Stable carbon isotope analysis measured using the CM-CRDS method can be effectively used to determine possible contamination of C4 sugar in honey samples. PCA analysis of δ^13^C together with the sugar profile information could potentially identify the addition of some C3 and C4 sugar sources. The authentic longan honey used in this study showed δ^13^C values of −26.26‰ and −25.68‰ for bulk honey and honey protein, respectively, using the CM-CRDS measurement technique. These δ^13^C values were in the same range as previously reported for Thai honey [30] and in a similar range reported for Thai and Taiwanese authentic longan honey [55].

In this study, we have demonstrated that δ^13^C measurements and melissopalynology can be implemented on the same set of honey samples to create a more stringent set of criteria for honey authentication. The proposed criteria were (1) δ^13^C_H_ not exceeding −22.00‰ based on the δ^13^C range of C3 plants, (2) Δδ^13^C_P-H_ of at least −1.00‰ using δ^13^C_P_ as the internal reference for δ^13^C_H_ measurements, (3) predominant pollen greater than 45% to ensure the predominant source of nectar, and (4) the predominant pollen species must match the claimed botanical origin of the honey sample. The first two criteria were applied to eliminate samples with possible C4 sugar adulteration. The latter two criteria were used to verify the botanical claim and monofloral honey consideration.

The above criteria set was applied to 34 beekeepers’ honey samples, comprising 26 samples of longan honey and 8 samples of other kinds of honey including wildflower honey, lychee honey, sunflower honey, and coffee blossom honey. δ^13^C measurements yielded a passing rate of 24 out of 34, or 70.6%, and melissopalynology yielded a passing rate of 13 out of 34, or 38.2%. When implemented together, the passing rate was reduced to 8 out of 34, or 23.5%, indicating increased stringency. When longan honey and the group of other kinds of honey were considered separately, similar passing rates were obtained. The passing rate for longan honey samples was 23.1%, and that of the group of other honey was 25.0%. Although other honey types were underrepresented in this study, it was demonstrated that the proposed criteria set could be applied to the longan honey and could likely be generalized and applied to other kinds of honey as well.

Longan honey is an economically important honey of Northern Thailand. Criteria that could ensure high-quality or premium-quality products can help to create value addition and allow these products to enter certain niche markets abroad. Of course, honey quality cannot depend only on δ^13^C and melissopalynology criteria; other criteria such as physicochemical properties and sensory quality must also be considered. Nonetheless, δ^13^C measurements and melissopalynology will certainly play an important role in creating high- or premium-quality longan honey products in Thailand, which can lead to an economically sustained apicultural practice among northern beekeepers.

## Figures and Tables

**Table 1:** δ^13^C values of a honey sample and its extracted proteins measured using EA/LC-IRMS, EA-IRMS, and CM-CRDS.

Method	δ^13^C_H_ ^1^	δ^13^C_P_ ^1^	∆δ^13^C_P-H_
EA/LC-IRMS	−26.43 ^a^	−25.57 ^b^	0.86
EA-IRMS	−26.51 ^a^	−26.00 ^a^	0.51
CM-CRDS	−26.26 ^a^	−25.68 ^b^	0.58

^1^ Values with the same superscripts (a and b) in a column indicate non-significant differences (*p* > 0.05) using Tukey’s Honestly Significant Difference (HSD) post hoc test.

**Table 2:** δ^13^C values of sugar syrups used as adulterants and the deduced C3/C4 plant sources.

Syrup Type	Labeled Source	δ^13^C (‰)	Deduced Plant Source ^1^
Cane syrup	Sugarcane	−13.04 ± 0.26	C4
Corn Syrup	Corn	−11.76 ± 0.09	C4
Golden syrup	Sugarcane	−12.78 ± 0.29	C4
Molasses	Sugarcane	−13.23 ± 0.10	C4
Maltose syrup	Rice and barley	−18.83 ± 0.21	C4 and C3
High-fructose syrup	Cassava	−26.92 ± 0.32	C3
Invert sugar	Sugar beet	−27.69 ± 0.29	C3
Rice syrup	Rice	−28.29 ± 0.20	C3
Glucose syrup	Cassava	−27.20 ± 0.21	C3

^1^ δ^13^C of C3 plants ranges from −32‰ to −22‰; δ^13^C of C4 plants ranges from −16‰ to −8‰ [18].

**Table 3:** δ^13^C values and classification results of honey samples from various botanical sources.

Honey Samples	Declared Origin	δ^13^C_H_ ^1,3^	δ^13^C_P_ ^1,3^	∆δ^13^C_P-H_	δ^13^C_H_ <−22.00‰ ^2^	∆δ^13^C_P-H_ >−1.00‰ ^2^
Longan A01	Longan blossom	−26.42 ± 0.12	−23.60 ± 0.06	2.82	A	A
Longan B01	Longan blossom	−22.89 ± 0.08	−22.76 ± 0.03	0.13	A	A
Longan C04	Longan blossom	−24.36 ± 0.15	−21.18 ± 0.13	3.18	A	A
Longan CP01	Longan blossom	−23.03 ± 0.28	−24.53 ± 0.12	−1.50	A	R
Longan CP02	Longan blossom	−25.19 ± 0.06	−25.17 ± 0.00	0.03	A	A
Longan CP03	Longan blossom	−23.72 ± 0.03	−24.66 ± 0.01	−0.94	A	A
Longan CP04	Longan blossom	−16.40 ± 0.41 *	−21.61 ± 0.03	−5.22	R	R
Longan CP05	Longan blossom	−24.96 ± 0.03	−23.21 ± 0.21	1.75	A	A
Longan CP06	Longan blossom	−26.34 ± 0.11	−25.23 ± 0.12	1.11	A	A
Longan CP07	Longan blossom	−20.87 ± 0.39	−22.15 ± 0.20	−1.28	R	R
Longan CP08	Longan blossom	−24.14 ± 0.26	−23.76 ± 0.12	0.38	A	A
Longan CP09	Longan blossom	−25.49 ± 0.08	−25.90 ± 0.14	−0.41	A	A
Longan CP10	Longan blossom	−25.23 ± 0.07	−25.02 ± 0.08	0.21	A	A
Longan F01	Longan blossom	−26.48 ± 0.22	−25.28 ± 0.18	1.20	A	A
Longan H01	Longan blossom	−27.11 ± 0.15	−24.60 ± 0.16	2.51	A	A
Longan H03	Longan blossom	−26.14 ± 0.14	−24.54 ± 0.05	1.60	A	A
Longan L01	Longan blossom	−27.68 ± 0.05	−25.47 ± 0.06	2.21	A	A
Longan N01	Longan blossom	−21.84 ± 0.04	−22.37 ± 0.16	−0.53	R	A
Longan P01	Longan blossom	−26.16 ± 0.01	−25.42 ± 0.44	0.73	A	A
Longan P02	Longan blossom	−24.22 ± 0.03	−25.28 ± 0.00	−1.06	A	R
Longan P03	Longan blossom	−25.66 ± 0.01	−24.69 ± 0.01	0.97	A	A
Longan P04	Longan blossom	−26.29 ± 0.02	−24.92 ± 0.02	1.37	A	A
Longan P05	Longan blossom	−25.57 ± 0.02	−24.78 ± 0.04	0.79	A	A
Longan P06	Longan blossom	−27.08 ± 0.08	−25.05 ± 0.12	2.03	A	A
Longan P07	Longan blossom	−26.56 ± 0.01	−25.07 ± 0.03	1.49	A	A
Longan P09	Longan blossom	−26.98 ± 0.07	−25.13 ± 0.00	1.85	A	A
Wildflower C06	Wild flowers	−15.64 ± 0.11 *	−21.15 ± 0.17	−5.51	R	R
Wildflower H04	Wild flowers	−22.93 ± 0.07	−24.44 ± 0.01	−1.51	A	R
Wildflower P08	Wild flowers	−26.00 ± 0.05	−25.64 ± 0.05	0.36	A	A
Lychee C05	Lychee blossom	−20.70 ± 0.20	−22.26 ± 0.05	−1.56	R	R
Lychee H02	Lychee blossom	−26.33 ± 0.07	−24.52 ± 0.05	1.81	A	A
Sunflower H01	Sunflower blossom	−27.07 ± 0.03	−26.98 ± 0.03	0.09	A	A
Sunflower K01	Sunflower blossom	−28.53 ± 0.19	−29.30 ± 0.07	−0.77	A	A
Coffee H01	Coffee blossom	−28.23 ± 0.09	−31.75 ± 0.01 *	−3.52	A	R

^1^ δ^13^C values are shown as mean ± SD from triplicate measurements. ^2^ “A” marks samples that passed the specified criteria and thus were accepted; “R” marks samples that failed and thus were rejected. ^3^ Asterisk “*” indicates a significant outlier based on Grubb’s test (*p* < 0.05).

**Table 4:** Pollen composition of 34 honey samples showing percentages of predominant, secondary, and minor pollen.

Honey Samples	Declared Origin	Predominant Pollen (>45%)	Secondary Pollen (16–45%)	Minor Pollen (3–15.9%)	Monofloral Honey as Claimed ^1^
Longan A01	Longan blossom	*Mimosa pudica* (72%)		*Bidens pilosa* L. (13%)	N
Longan B01	Longan blossom	*Mimosa pudica* (46%)	*Dimocarpus longan* L. (41%)		N
Longan C04	Longan blossom	*Dimocarpus longan* L. (50%)	*Mimosa pudica* (18%)		Y
Longan CP01	Longan blossom		*Dimocarpus longan* L. (41%) *Mimosa pudica* (28%)		N
Longan CP02	Longan blossom	*Dimocarpus longan* L. (46%)	*Mimosa pudica* (21%)		Y
Longan CP03	Longan blossom	*Dimocarpus longan* L. (50%)	*Mimosa pudica* (31%)		Y
Longan CP04	Longan blossom	*Dimocarpus longan* L. (48%)	*Mimosa pudica* (35%)		Y
Longan CP05	Longan blossom		*Dimocarpus longan* L. (44%) *Mimosa pudica* (36%)		N
Longan CP06	Longan blossom		*Dimocarpus longan* L. (44%) *Mimosa pudica* (28%)		N
Longan CP07	Longan blossom	*Mimosa pudica* (52%)	*Dimocarpus longan* L. (29%)		N
Longan CP08	Longan blossom		*Mimosa pudica* (38%) *Mimosa pigra* (28%) *Dimocarpus longan* L. (25%)		N
Longan CP09	Longan blossom	*Mimosa pigra* (82%)		*Mimosa pudica* (15%)	N
Longan CP10	Longan blossom	*Dimocarpus longan* L. (66%)	*Leucaena leucocephala* L. (18%)		Y
Longan F01	Longan blossom	*Mimosa pudica* (52%)	*Bidens pilosa* L. (21%)		N
Longan H01	Longan blossom	*Mimosa pudica* (69%)	*Dimocarpus longan* L. (18%)		N
Longan H03	Longan blossom		*Dimocarpus longan L*. (41%)	*Mimosa pudica* (15%)	N
Longan L01	Longan blossom	*Dimocarpus longan L*. (52%)	*Mimosa pudica* (20%)		Y
Longan N01	Longan blossom	*Mimosa pudica* (60%)	Salicaceae (29%)		N
Longan P01	Longan blossom		*Dimocarpus longan* L. (45%) *Mimosa pudica* (34%)		N
Longan P02	Longan blossom		*Mimosa pudica* (32%) *Dimocarpus longan* L. (29%)		N
Longan P03	Longan blossom	*Mimosa pudica* (47%)	*Dimocarpus longan* L. (35%)		N
Longan P04	Longan blossom		*Dimocarpus longan* L. (40%) *Mimosa pudica* (37%)		N
Longan P05	Longan blossom	*Mimosa pudica* (66%)	*Dimocarpus longan* L. (18%)		N
Longan P06	Longan blossom	*Dimocarpus longan* L. (54%)	*Mimosa pudica* (27%)		Y
Longan P07	Longan blossom		*Dimocarpus longan* L. (37%) *Mimosa pudica* (25%)		N
Longan P09	Longan blossom	*Dimocarpus longan* L. (55%)	*Mimosa pudica* (24%)		Y
Wildflower C06	Wild flowers	*Mimosa pudica* (81%)		*Bidens pilosa* L. 7%	Y
Wildflower H04	Wild flowers	*Mimosa pudica* (61%)		Fabaceae 12%	Y
Wildflower P08	Wild flowers	*Mimosa pudica* (62%)	*Dimocarpus longan* L. (22%)		Y
Lychee C05	Lychee blossom		*Mimosa pudica* (31%) *Litchi chinensis* (25%)		N
Lychee H02	Lychee blossom		*Litchi chinensis* (26%) Polygonaceae family (21%)		N
Sunflower H01	Sunflower blossom		*Helianthus annuus* (37%) Salicaceae family (26%)		N
Sunflower K01	Sunflower blossom	*Helianthus annuus* (88%)		Fabaceae 7%	Y
Coffee H01	Coffee blossom	*Coffea* (93%)		Poaceae (3%)	Y

^1^ The letter Y designates samples that passed the monofloral honey criterion; the letter N designates samples that failed. Predominant pollen must be >45% to pass the criterion.

**Table 5:** Authentication consideration of 34 honey samples.

Honey Samples	δ^13^C Criteria ^1^	Monofloral Honey Criteria ^2^	Final Consideration ^3^
Longan A01	A	N	F
Longan B01	A	N	F
Longan C04	R	Y	F
Longan CP01	R	N	F
Longan CP02	A	Y	P
Longan CP03	A	Y	P
Longan CP04	R	Y	F
Longan CP05	A	N	F
Longan CP06	A	N	F
Longan CP07	R	N	F
Longan CP08	A	N	F
Longan CP09	A	N	F
Longan CP10	A	Y	P
Longan F01	A	N	F
Longan H01	A	N	F
Longan H03	A	N	F
Longan L01	A	Y	P
Longan N01	R	N	F
Longan P01	A	N	F
Longan P02	R	N	F
Longan P03	A	N	F
Longan P04	A	N	F
Longan P05	A	N	F
Longan P06	A	Y	P
Longan P07	A	N	F
Longan P09	A	Y	P
Wildflower C06	R	Y	F
Wildflower H04	R	Y	F
Wildflower P08	A	Y	P
Lychee C05	R	N	F
Lychee H02	A	N	F
Sunflower H01	A	N	F
Sunflower K01	A	Y	P
Coffee H01	R	Y	F

^1^ “A” marks samples that passed the specified criteria and were thus accepted; “R” marks samples that failed and were thus rejected. Both the C3 plant δ^13^C range and ∆δ^13^C_P-H_ were considered. ^2^ “Y” designates samples that passed the monofloral honey criterion; “N” designates samples that failed. ^3^ “P” designates samples that passed the final consideration; “F” designates samples that failed.

## Data Availability

The original contributions presented in the study are included in the article and Supplementary Material. Further inquiries can be directed to the corresponding author.

## Supplementary Materials

The following supporting information can be downloaded at: https://www.mdpi.com/article/10.3390/foods14223850/s1, Table S1: Linear regression statistics based on δ^13^C_H_ values from CM-CRDS (y) and from EA-IRMS (x) assuming y = *a*x; Table S2: Linear regression statistics based on δ^13^C_P_ values from CM-CRDS (y) and from EA-IRMS (x) assuming y = *a*x; Table S3: Reproducibility of δ^13^C measurements by CM-CRDS. δ^13^C_H_ and δ^13^C_P_ of a honey sample designated ‘Sample C’ was measured in nine separate preparations using CM-CRDS; Table S4: Factor loading results obtained from the principal component analysis (PCA) of deliberately adulterated honey samples.

## Abbreviations

The following abbreviations are used in this manuscript:

SCIRA	Stable carbon isotope ratio analysis
EA/LC-IRMS	Elemental analyzer/liquid chromatography–isotope ratio mass spectrometer
EA-IRMS	Elemental analyzer–isotope ratio mass spectrometer
CM-CRDS	Combustion module–cavity ring down spectroscopy
δ^13^C_H_	δ^13^C of bulk honey
δ^13^C_P_	δ^13^C of honey protein fraction
δ^13^C_P-H_	δ^13^C of protein fraction minus δ^13^C of bulk honey
HPLC	High-performance liquid chromatography
