# Supplementary material for: A Synergistic Approach Combining Stable Carbon Isotope Ratio Analysis and Melissopalynology for the Authentication of Honey from Thailand
Source: Foods. 2025 Nov 11;14(22):3850. doi: 10.3390/foods14223850 (PMC12651881; doi:10.3390/foods14223850)
Supplement: Supplementary file 1 [file foods-14-03850-s001.zip › foods-3935682-supplementary.pdf]

Supplementary Materials

Table S1. Linear regression statistics based on $\delta^{13}C_H$ values from CM-CRDS (y) and from EA-IRMS (x) assuming y = ax.

Regression Statistics	
Multiple R	0.999968
R Square	0.999937
Adjusted R Square	0.969634
Standard Error	0.200972
Observations	34

ANOVA

	df	*SS*	*MS*	*F*	*Significance F*
Regression	1	21148.75	21148.75	523615.2	5.29E-69
Residual	33	1.332866	0.04039		
Total	34	21150.09			

	Coefficients	*Standard Error*	*t Stat*	*P-value*	*Lower 95%*	*Upper 95%*	*Lower 95.0%*	*Upper 95.0%*
Intercept	0	#N/A	#N/A	#N/A	#N/A	#N/A	#N/A	#N/A
X Variable 1	1.000022	0.001382	723.6126	6.77E-71	0.997211	1.002834	0.997211	1.002834

Table S2. Linear regression statistics based on $\delta^{13}C_P$ values from CM-CRDS (y) and from EA-IRMS (x) assuming y = ax.

Regression Statistics	
Multiple R	0.999663
R Square	0.999325
Adjusted R Square	0.969022
Standard Error	0.654699
Observations	34

ANOVA

	df	*SS*	*MS*	*F*	*Significance F*
Regression	1	20952.43	20952.43	48882.21	1.58E-52
Residual	33	14.14482	0.428631		
Total	34	20966.57			

	Coefficients	*Standard Error*	*t Stat*	*P-value*	*Lower 95%*	*Upper 95%*	*Lower 95.0%*	*Upper 95.0%*
Intercept	0	#N/A	#N/A	#N/A	#N/A	#N/A	#N/A	#N/A
X Variable 1	1.004448	0.004543	221.0932	6.59E-54	0.995205	1.013691	0.995205	1.013691

Table S3. Reproducibility of $\delta^{13}C$ measurements by CM-CRDS. $\delta^{13}C_H$ and $\delta^{13}C_P$ of a honey sample designated 'Sample C' was measured in nine separate preparations using CM-CRDS.

Trial No.	$\delta^{13}C_H$ (‰)	$\delta^{13}C_P$ (‰)
**1**	-25.30	-25.51
**2**	-25.29	-25.36
**3**	-25.23	-25.27
**4**	-25.30	-25.02
**5**	-25.24	-25.55
**6**	-25.30	-25.50
**7**	-25.29	-25.44
**8**	-25.30	-25.50
**9**	-25.24	-25.44
**Mean**	-25.28	-25.40
**SD**	0.03	0.17
**%RSD**	0.12	0.65

Table S4. Factor loading results obtained from the principal component analysis (PCA) of deliberately adulterated honey samples.

Indicator Variables	Factor Loading	
	PC1	PC2
Maltose	-0.882	-0.197
Glucose	0.748	-0.404
Fructose	0.910	-0.304
$\delta^{13}C_H$	0.025	0.655
$\delta^{13}C_P$	-0.494	0.647
Eigenvalues	2.94	1.24
Explained variance (%)	58.8	24.8
Cumulative variance (%)	58.8	83.6
